# Potential causes of early death among admitted newborns in a rural Tanzanian hospital

**DOI:** 10.1371/journal.pone.0222935

**Published:** 2019-10-02

**Authors:** Robert Moshiro, Jeffrey M. Perlman, Paschal Mdoe, Hussein Kidanto, Jan Terje Kvaløy, Hege L. Ersdal

**Affiliations:** 1 Faculty of Health Sciences, University of Stavanger, Stavanger, Norway; 2 Department of Paediatrics and Child Health, Muhimbili National Hospital, Dar es Salaam, Tanzania; 3 Department of Pediatrics, Weill Cornell Medicine, New York, NY, United States of America; 4 Department of Obstetrics and Gynecology, Haydom Lutheran Hospital, Manyara, Tanzania; 5 School of Medicine, Aga Khan University, Dar es Salaam, Tanzania; 6 Research Department, Stavanger University Hospital, Stavanger, Norway; 7 Department of Mathematics and Physics, University of Stavanger, Stavanger, Norway; 8 Department of Anesthesiology and Intensive Care, Stavanger University Hospital, Stavanger, Norway; Agha Khan University, UNITED REPUBLIC OF TANZANIA

## Abstract

**Background:**

Approximately 40,000 newborns die each year in Tanzania. Regional differences in outcome are common. Reviewing current local data, as well as defining potential causal pathways leading to death are urgently needed, before targeted interventions can be implemented

**Objective:**

To describe the clinical characteristics and potential causal pathways contributing to newborn death and determine the presumed causes of newborn mortality within seven days, in a rural hospital setting.

**Methods:**

Prospective observational study of admitted newborns born October 2014–July 2017. Information about labour/delivery and newborn management/care were recorded on data collection forms. Causes of deaths were predominantly based on clinical diagnosis.

**Results:**

671 were admitted to a neonatal area. Reasons included prematurity n = 213 (32%), respiratory issues n = 209 (31%), meconium stained amniotic fluid with respiratory issues n = 115 (17%) and observation for < 24 hours n = 97 (14%). Death occurred in 124 infants. Presumed causes were birth asphyxia (BA) n = 59 (48%), prematurity n = 19 (15%), presumed sepsis n = 19 (15%), meconium aspiration syndrome (MAS) n = 13 (10%) and congenital abnormalities n = 14 (11%). More newborns who died versus survivors had oxygen saturation <60% on admission (37/113 vs 32/258; *p*≤0.001) respectively. Moderate hypothermia on admission was common i.e. deaths 35.1 (34.6**–**36.0) vs survivors 35.5 (35.0**–**36.0)°C (*p*≤0.001). Term newborns who died versus survivors were fourfold more likely to have received positive pressure ventilation after birth i.e. 4.57 (1.22–17.03) (*p*<0.02).

**Conclusion:**

Intrapartum-related complications (BA, MAS), prematurity, and presumed sepsis were the leading causes of death. Intrapartum hypoxia, prematurity and attendant complications and presumed sepsis, are major pathways leading to death. Severe hypoxia and hypothermia upon admission are additional contributing factors. Strategies to identify fetuses at risk during labour e.g. improved fetal heart rate monitoring, coupled with timely interventions, and implementation of WHO interventions for preterm newborns, may reduce mortality in this low resource setting.

## Introduction

In Tanzania, approximately 40,000 newborns die annually due to three major causes; birth asphyxia (BA), prematurity and presumed infections [1,2]. Approximately half of newborn deaths occur within the first 24 hours, and two thirds by the seventh day [3]. Early newborn deaths result mainly from intrapartum complications and immediate postnatal problems including apnea, meconium and hypothermia, which may be prevented or ameliorated by timely interventions.

BA defined as a 5-minute Apgar score <7 and lack of spontaneous respirations after birth, accounts for approximately 0.7 million global deaths [4]. Specifically, in Tanzania, BA accounts for 27 to 45 percent of neonatal deaths; this range in BA reflects regional differences in the country [2, 5, 6]. Globally, prematurity and its attendant complications is the leading cause of neonatal mortality [5]. In Tanzania, approximately 11 percent of newborns are born premature i.e. <37 weeks but they account for 23–35 percent of newborn deaths [1,2]. While in many resource-replete countries, the lower limit of viability approximates 24 weeks of gestation [7], low-resource countries still struggle to prevent premature deaths even at 28 weeks [8]. Recently, the WHO recommended several interventions including administration of antenatal corticosteroids to women at imminent risk of premature labour, and early initiation of continuous positive airway pressure (CPAP) for newborns with respiratory distress syndrome (RDS), geared towards reducing preterm mortality in low-resource settings [9]. A third leading cause of death are infections, which contribute to anywhere from 10 to 30 percent of newborn mortality in Tanzania [1, 2]. However, the diagnosis of infection is often presumed because blood cultures and other ancillary tool are not consistently obtained. Prior to implementation of any potential intervention current local data are needed, with a specific focus on potential causal pathways leading to mortality, in order to facilitate targeted specific interventions. The objectives of this study were to: (1) determine the presumed causes of newborn mortality in a rural hospital setting, and (2) describe the clinical characteristics and potential causal pathways contributing to newborn death within seven days.

## Materials and methods

From October 2014 to July 2017, a prospective observational study was conducted at Haydom, a rural hospital in Northern Tanzania, of consecutively admitted newborns followed until discharge or death within the first seven days. Ethical approval was granted by the National Institute for Medical Research in Tanzania (Ref. NIMR/HQ/R.8a/Vol.IX/1434) and the Regional Committee for Medical and Health Research Ethics in Norway (Ref.2013/110). Informed consent was not required by the ethical committees because the study was descriptive.

HLH has approximately 4500 deliveries annually, which is about 53% of deliveries in the catchment area. Less than 10% give birth in other facilities and the remainder gives birth at home. The hospital provides emergency obstetric services 24 hours a day with a caesarean section rate of 22% [10]. Deliveries and newborn resuscitations were mainly conducted by 18–22 midwives with three shifts. Midwives were trained in Helping Babies Breathe [11] and short ventilation training sessions were conducted on a weekly basis with full HBB refresher training twice yearly. The management of newborns born through meconium stained amniotic fluid (MSAF) focused on clearing the oro-pharynx with a bulb suction, when meconium was present, followed by positive pressure ventilation for the non-breathing infant.

The hospital’s newborn area accommodates 10–15 newborns with one general practitioner in charge of the neonatal ward, supervising intern doctors. Admission criteria include prematurity, 5-minute Apgar score <7, fever (temperature >38°C), signs of respiratory compromise, i.e. intercostal, subcostal retractions or grunting and infants with congenital abnormalities. Premature newborns are nursed under shared radiant warmer and term newborns are nursed in locally made ‘baby cots’. Interventions include intravenous fluids, intravenous antibiotics, oxygen therapy using oxygen concentrators, and phototherapy, as clinically indicated. Stable newborns are allowed to breastfeed, if not, mothers express milk and it is administered either via an oro-gastric tube or via a cup. Stable premature newborns of <1800 grams are nursed skin to skin until they attain a minimum discharge weight of 1800 grams. Provisional diagnoses and presumed causes of death were assigned by the attending doctor during the ward round.

Trained research assistants (*n* = 14) observed and recorded every delivery in the labour ward and theatre on a data collection form. This form contained antenatal and perinatal information. Information related to admitted newborns was captured using a second data collection form. Inclusion criteria were all newborns delivered at HLH and were alive at the time of admission to the newborn area. Exclusion criteria included: all newborns delivered outside HLH, and newborns who died in the delivery room within 30 minutes of birth. Newborns who died in the delivery room were excluded as they have been reported previously [12]. More specific definitions are noted in Table 1.

**Table 1:** Specific definitions used to define conditions.

	Definition
Gestational age	Self-report of the last normal menstrual period and distance measured from symphysis pubis to the fundus
Prematurity	Gestational age <37 weeks
Low birth weight	Birth weight <2500 grams.
Presumed Neonatal sepsis	One or more clinical signs of bacterial infection: pallor, poor perfusion, bradycardia, apnea, tachypnea (>60 breaths/minute), dyspnoea (grunting, nasal flaring, retractions), temperature instability (≥38°C or <36°C), difficulty feeding and distended abdomen.
Pneumonia	Difficulty in breathing, tachypnea (respiratory rate >60 breaths/minute) without history of birth asphyxia.
Birth asphyxia	1) Failure to initiate spontaneous breathing and/or 5-minute Apgar score <7 in addition to clinical evidence of encephalopathy. 2) Gestational age <32 weeks with a history of suspected intrapartum related hypoxia (abnormal fetal heart rate, labour complications, no respiratory efforts or the need for positive pressure ventilation)
Respiratory distress syndrome	Considered in a preterm baby with chest retractions within two hours after delivery with exacerbation over the first 24–48 hours of life, followed by a stable phase till 72 hours, then improvement between the 3^rd^ and 6^th^ day.
Meconium aspiration syndrome	Onset of respiratory distress immediately after birth or few hours, with history of meconium stained amniotic fluid, meconium around the oro-pharynx or meconium stained skin.
Meconium stained amniotic fluid	Amniotic fluid stained with thick meconium
Hypoxic-ischaemic encephalopathy	Clinical diagnosis characterized as mild, moderate and severe using the Thompson scoring system

### Initial management of admitted newborns

Management of newborns followed the principles outlined in the WHO Essential Newborn Care guidelines which included routine administration of intramuscular Vitamin K injection (1 mg stat for term and 0.5 mg for preterm) to all admitted newborns [13]. Axillary temperature and blood glucose measurements were obtained using a digital thermometer and commercial glucometer, respectively, within 30 minutes of admission. Hypoglycemia, defined as blood glucose below 2.5 mmol/L [14], was treated with intravenous bolus 3 mls/kg 10% dextrose followed by feeding or maintenance with 10% dextrose. Seizures were treated with intravenous phenobarbital at a loading dose of 20 mg/kg, followed by maintenance dose of 5 mg/kg/day. Newborns with history of BA were categorized clinically as either mild, moderate and severe hypoxic ischaemic encephalopathy using the Thompson scoring system [15]. Antibiotics (ampicillin 50 mg/kg/day and gentamycin 4 mg/kg/day) were given to at risk newborns, i.e. premature newborns, infants with presumed MAS and/or BA, for at least 48 hours because of the inability to exclude infections. If sepsis was suspected based on clinical signs [16, 17], the antibiotics were changed to ceftriaxone 50 mg/kg/day for 10 days. Continuous positive airway pressure (CPAP) and intubation was not available to assist infants with respiratory difficulty. Radiological investigations such as chest x-rays and cranial ultrasounds were infrequently obtained because of difficulties transporting unstable newborns to the radiology department. Blood cultures, full blood counts and C-reactive protein measurements were rarely available.

#### Data analysis

The Chi-square or Fisher exact test was used to test for differences between categorical variables, as appropriate. The Mann-Whitney U test was used to test for differences between groups for continuous variables when test for normality showed that the data where not normally distributed. Logistic regression was used for modelling the impact of risk factors for death among admitted newborns. First a stepwise backward selection method with the retention of predictors with *p*<0.05 was used, and afterwards excluded variables were re-entered one by one and kept if found significant. The results are presented as Odds Ratio (OR) with 95% confidence intervals (CIs). Variables obtained on initial assessment during admission were not used because of missing data. Statistical analyses were performed using SPSS (IBM SPSS Statistics for Windows, version 22.0; IBM Corp., Armonk, N.Y. USA).

## Results

**Fig 1:**
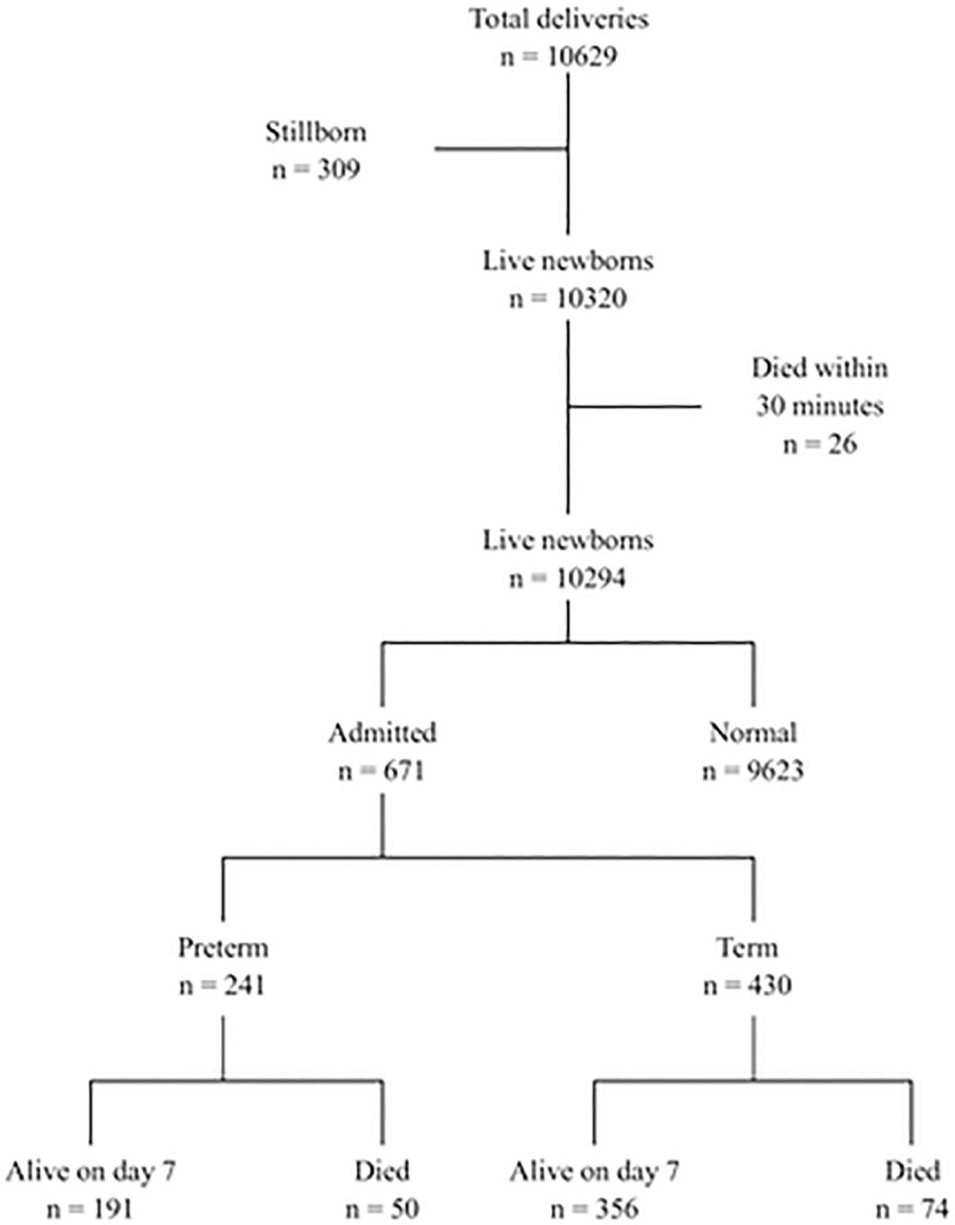
Flow diagram of newborn delivered and outcome after 7 days.

During the study period, there were 10629 deliveries, 10320 were live newborns of birth weight 3311±529 grams and GA 38.8±1.8 weeks, 309 were stillborn and 26 newborns died within 30 minutes (the latter newborns died from BA) [12]. Of the remaining 10294 newborns, 9623 were normal and stayed with the mother, and 671 were admitted to the neonatal area (Fig 1). Of the admitted newborns, a total of 124/671 (18%) died within seven days; of these 61 (49%) within 24 hours, an additional 38 (31%) within 72 hours and 25 beyond 72 hours of life.

### Delivery room management (n = 10320)

At delivery 2785/10320 (27%) were stabilized with stimulation and/or suctioning and 691 (6.7%) of these newborns were ventilated immediately after delivery. MSAF was present in 2561 (25%) of all live newborns.

### Characteristics of admitted newborns (n = 671)

Of the 671 admissions, 241 (36%) were premature and 297 (44%) were of low birth weight. A total of 314 (46%) of the admitted newborns were ventilated in the delivery room; of these 17% were premature. Reasons for admission included complications of prematurity *n* = 213 (32%), respiratory issues *n* = 209 (31%), MSAF with respiratory issues *n* = 115 (17%), infants admitted for observation <24 hour (e.g. post-caesarean section, infants with weight above 3.8 kg) *n* = 97 (14%), and other indication 37 (5%).

### Presumed causes of death *(n =* 124)

**Fig 2:**
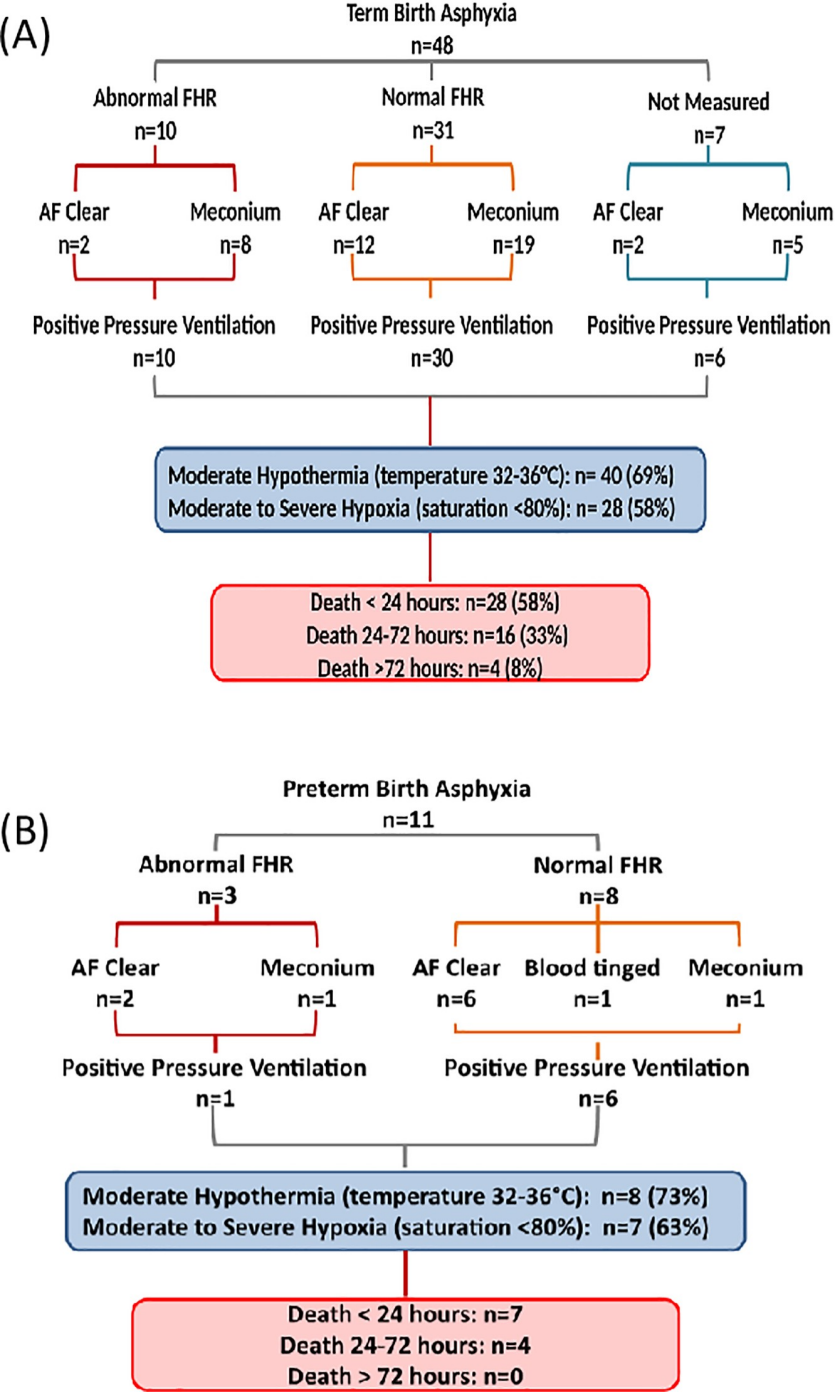
Perinatal characteristics, hypothermia and hypoxia and timing of death in infants with birth asphyxia. **(A) Term infants. (B) Preterm infants.** Abbreviations: FHR, foetal heart rate; AF, amniotic fluid.

**Fig 3:**
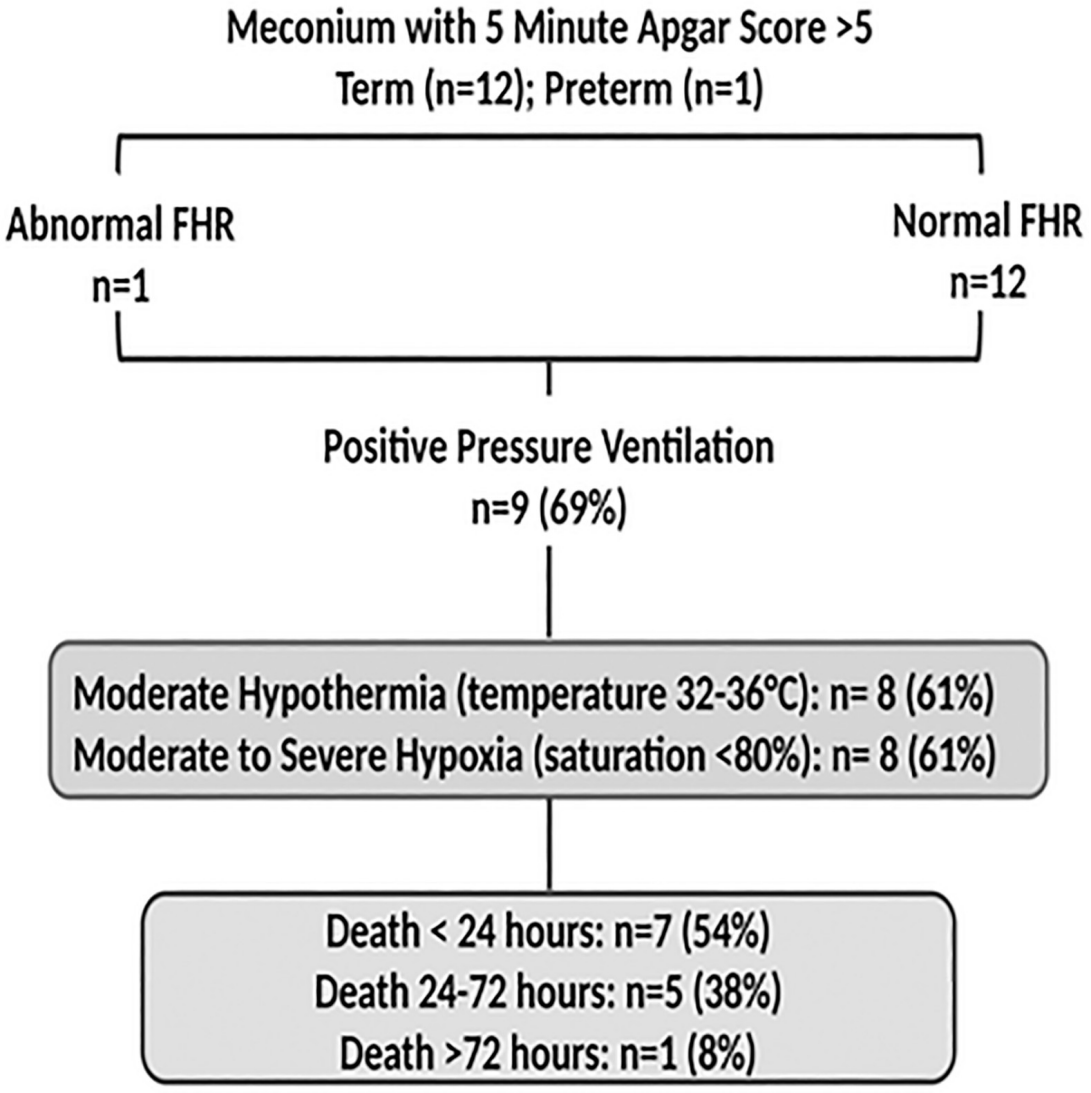
Perinatal characteristics, presence of hypothermia and hypoxia in infants with meconium aspiration syndrome. Abbreviations: FHR, foetal heart rate.

**Fig 4:**
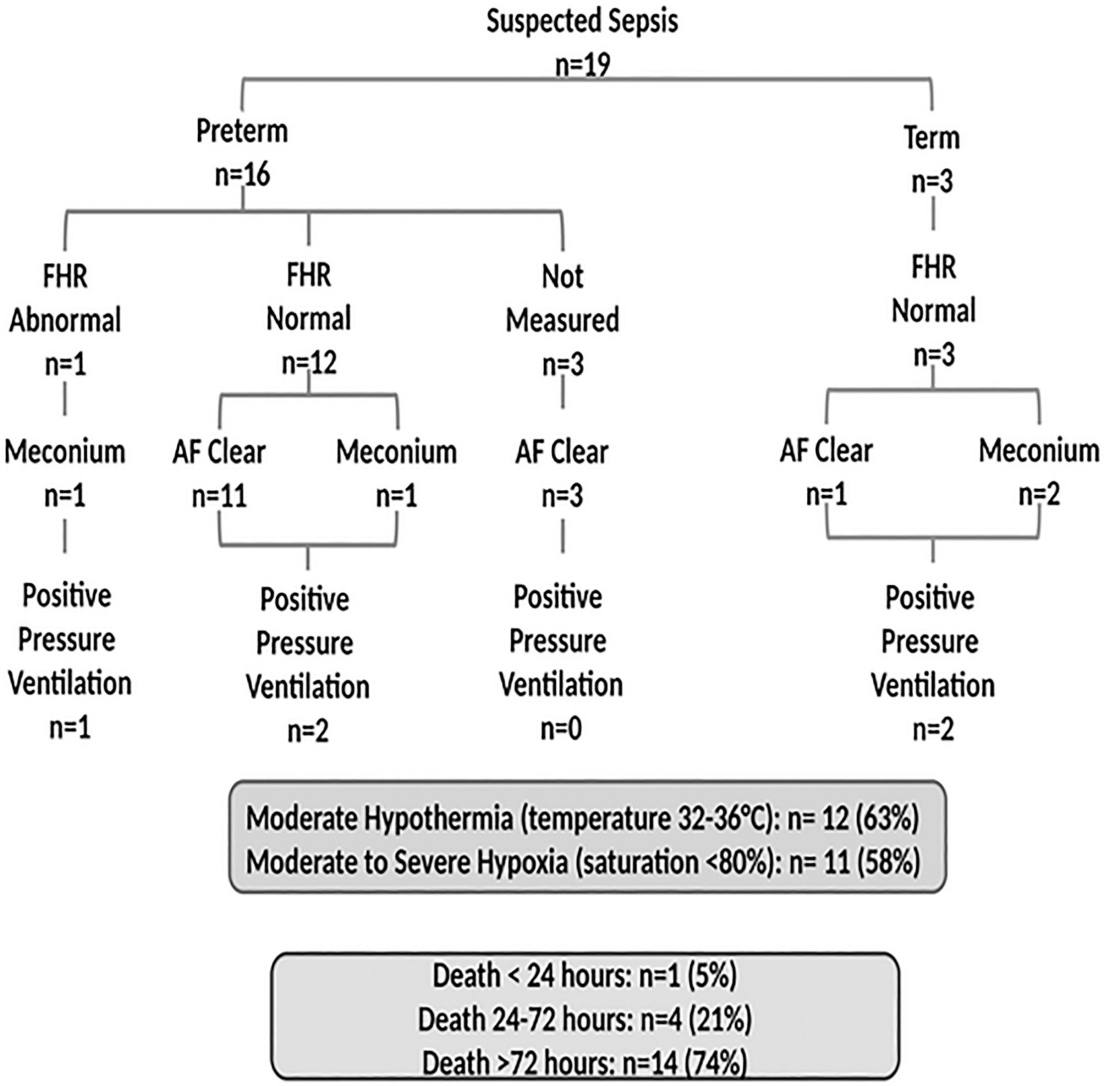
Perinatal characteristics, presence of hypothermia and hypoxia and timing of death in preterm infants. Abbreviations: FHR, foetal heart rate; AF, amniotic fluid.

**Fig 5:**
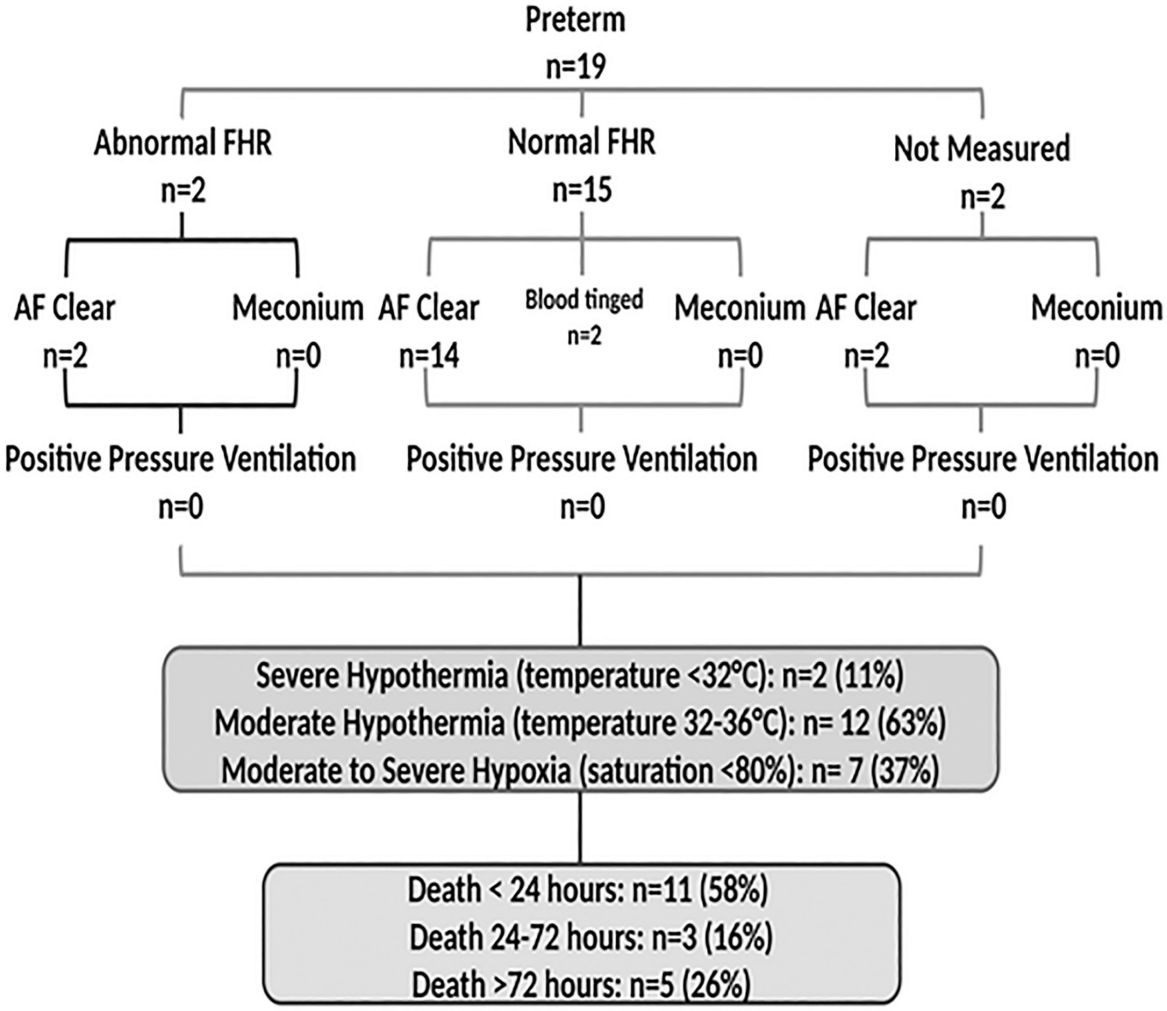
Perinatal characteristics, hypothermia and hypoxia and timing of death in infants with presumed/suspect sepsis. Abbreviations: FHR, foetal heart rate; AF, amniotic fluid.

The presumed causes of death included BA (n = 59), complications related to MSAF (*n* = 13), prematurity (*n* = 19), presumed/suspect infection (*n* = 19) and secondary to complication related to congenital anomalies (*n* = 14). Each is briefly reviewed below:

1. BA (*n* = 59) (47%) was the leading cause of death which included 11 (22%) premature newborns (Fig 2). Labour was complicated by an abnormal fetal heart rate in 13 (22%) cases. In 7(12%) the foetal heart rate was not measured. Delivery was via emergency caesarean section in 30 (51%) cases and in 34 (58%) there was associated MSAF (Fig 2). Positive pressure ventilation was applied in the delivery room in 54 (92%) newly borns. Upon admission to the neonatal ward, moderate hypothermia (temperature 32–36°C) was noted in 40 (69%) and moderate to severe hypoxia (saturation <80%) in 28 (58%) newborns (Fig 2). Hypoxic-ischemic encephalopathy (HIE) developed in 28 (48%) newborns, was severe in 17 (29%) and moderate in 11 (19%) of these newborns. Seizures were noted in 16 (27%) newborns. Death occurred within 24 hours in 35 (60%), and between 24 and 72 hours in 16 (33%) newborns.

2. MSAF was present in 254/671 (38%) of newborns; 122 (48%) cases were categorized as thick. There were 51 (20%) newborns with MSAF who died including 34 (13%) with BA (see above), 4 newborns with presumed/suspect sepsis and 13 (5%) additional newborns who died secondary to presumed MAS (Fig 3). For the latter newborns, the fetal heart rate during labour was normal in 12 (92%) cases. The MSAF was categorized as thick (n = 9) (69%) and delivery was via emergency CS in 9 (69%) cases. Positive pressure ventilation was applied in 9 (69%) and the 5-minute Apgar score was > 7 in all 13 newly borns. Moderate hypothermia was noted in 8/13 (61%) and moderate to severe hypoxia in 8 (61%) newborns. Death occurred within 24 hours in 7 (54%) and between 24 and 72 hours in 5 (38%) newborns.

3. Prematurity and its attendant complications contributed to 19 (15%) of all newborn deaths. Upon admission, all 19 had a normal foetal heart rate and the heart rate continued to be normal during labour in 17/19 cases; in 2 cases the foetal was not measured (Fig 4). Delivery was vaginal in 14 (74%) and via emergent caesarean section in 4 (21%) newborns. No newborn received positive pressure ventilation in the delivery room. Moderate to severe hypothermia was noted in 14 (74%) and moderate to severe hypoxia in 7 (37%) newborns. Complications of prematurity that led to death included respiratory distress syndrome (*n* = 11) (including 8 who died within 72 hours), recurrent apnoea (*n* = 2), necrotizing enterocolitis (*n* = 1), haemorrhage due to presumed Vitamin K deficiency (*n* = 1) and two newborns admitted with severe hypothermia (temperature <32⁰C). Death occurred within 24 hours in 11 (58%), between 24 and 72 hours in 3 (16%) and beyond 72 hours in 5 (26%) newborns.

4. Presumed/ suspect sepsis contributed to 19 (15%) deaths; 16 (84%) were premature. The foetal was normal in 15 (94%) neonates; in 3 cases the heart rate was not measured (Fig 5). Positive pressure ventilation was applied in 5 (26%) newly borns and in all cases the 5-minute Apgar score was ≥7. Moderate to severe hypothermia was noted in 12 (63%) and moderate to severe hypoxia in 11 (58%) newborns. Three of the newborns had presumed pneumonia. Death occurred within 24 hours in 1 (5%), between 24 and 72 hours in 4 (21%) and beyond 72 hours in 14 (74%) newborns.

5. Congenital abnormalities (*n* = 14) were associated with early deaths. These included presumed congenital heart disease (*n* = 3), anencephaly (*n* = 1), gastroschisis (*n* = 1), presumed congenital Rubella syndrome (*n* = 1), trachea-oesophageal fistula (*n* = 1), prune belly syndrome (*n* = 1), Hydrops fetalis (*n* = 1) and Down syndrome (*n* = 1). Four newborns had dysmorphic features which could not be linked to a definitive syndrome.

### Characteristics of newborns who died versus survivors

Newborns who died (*n* = 124) compared to those who survived (*n* = 547), were more likely to be male (*p* = 0.019), have a lower 1-minute (*p* = 0.01) and 5-minute Apgar score <7 (*p* = 0.001), have a lower admission temperature (*p*<0.001), lower oxygen saturation (*p*<0.001), more likely to receive supplemental oxygen administration (*p*<0.001), and higher random blood glucose levels (*p* = 0.04) (Table 2). Mode of delivery and indication for caesarean section were the same between those who died and survivors.

**Table 2:** Comparison of perinatal characteristics among newborns who died versus survivors within first 7 days.

	Dead *n* = 124	Survived *n* = 547	*P*-value
Birth weight (grams) (IQR)	2522 (1740–3010)	2640 (1930–3200)	0.07^a^
Gestational age (weeks) (IQR)	37 (32–40)	37 (34–40)	0.55 ^a^
Antenatal care attendance			
Yes	122 (98)	539 (99)	
No	2 (2)	7 (1)	0.77^c^
Antenatal complications			
Yes	6 (5)	30 (5)	
No	118 (95)	516 (95)	0.67^b^
FHR during labour			
Normal	92 (75)	424 (78)	
Abnormal/not detected	16 (13)	60 (11)	
Not measured	15 (12)	62 (11)	0.76^b^
Amniotic fluid colour			
Normal	69 (56)	333 (61)	
Meconium stained	52 (42)	202 (37)	
Blood stained	3 (2)	12 (2)	0.56^b^
Obstetric complication			
Yes	16 (13)	81 (15)	
No	108 (87)	465 (85)	0.58^b^
Mode of delivery			
Vaginal	71 (58)	335 (61)	
Caesarean section	52 (42)	211 (39)	0.10^b^
Gender			
Male	76 (61)	286 (52)	
Female	47 (39)	260 (48)	0.019^b^
PPV attempted			
Yes	73 (59)	241 (44)	
No	51 (41)	306 (67)	0.003^b^
Apgar score at 1 minute			
<7	69 (56)	178 (33)	
≥7	55 (44)	369 (67)	0.001^b^
Apgar score at 5 minute			
<7	39 (32)	48 (9)	
≥7	85 (68)	499 (91)	<0.001^b^
Oxygen saturation on admission			
<60%	37 (33)	32 (12)	
60–89%	52 (46)	94 (36)	
≥90%	24 (21)	132 (51)	<0.001
Initial assessment on admission			
Temperature (C)	35.1 (34.6–36.0)	35.5 (35.0–36.0)	<0.001^a^
Heart rate (beats/minute)	143 (130–158)	140 (130–154)	0.48^a^
Need for oxygen therapy (n)	93 (92)	132 (46)	<0.001^b^
Random blood glucose (mmol/l)	4.2 (2.4–5.5)	3.5 (2.3–4.6)	0.04^a^

Data are presented as *n* (%) unless otherwise stated

FHR, fetal heart rate; C, Celsius; PPV, positive pressure ventilation

^a^ Mann-Whitney

^b^ Chi-square

^c^Fisher-exact test

Additional analysis of term newborns only (excluding congenital malformations) was performed. Term newborns who died (*n* = 63) compared to those who survived (n = 353), were more likely to have thick meconium (p<0.001), have a 5 minute Apgar score <7 (*p*<0.001), have received positive pressure ventilation in the delivery room (*p*<0.001) and have severe hypoxia during admission (*p*<0.001) (Table 3).

**Table 3:** Comparison of perinatal characteristics among term newborns who died versus survivors within first 7 days.

	Dead	Survived	*P-*value^a^
	n = 63	n = 353	
FHR during labour (n = 382)			
Normal	45 (80)	285 (87)	
Abnormal/not detected	11 (20)	41 (13)	0.115
Amniotic fluid colour (n = 306)			
Normal	17 (38)	173 (66)	
Thick meconium stained	28 (62)	88 (34)	<0.001
Apgar score at 5 minute (n = 416)			
<7	33 (52)	38 (11)	
≥7	30 (48)	315 (89)	<0.001
PPV (n = 227)			
<60%	22 (39)	21 (12)	
60–89%	19 (34)	66 (39)	
>90%	15 (27)	84 (49)	<0.001
HIE score			
Mild	11 (30)	102 (94)	
Moderate	9 (24)	7 (6)	
Severe HIE	17 (46)	0 (0)	<0.001^b^

Data is presented as *n* (%) unless otherwise stated

FHR, fetal heart rate; PPV, positive pressure ventilation; HIE, hypoxic ischemic encephalopathy

^a^Chi-square test unless stated otherwise

^b^Fisher-exact test

Logistic regression model of independent predictors of death for all admitted newborns and a subgroup of term newborns (excluding newborns with congenital malformations) is shown in Tables 4 and 5.

**Table 4:** Logistic regression model of independent predictors of death among all admitted newborns.

Variables	Bivariate analysis	*P-value*	Multivariable analysis with all variables into the model	*P-value*	Multivariable analysis after backward LR elimiation	*P-value*
	OR (95%CI)		AOR (95%CI)		AOR (95%CI)	
Gestational age (weeks)	97 (0.92–1.03)	0.34	0.99 (0.87–1.13)	0.91		
Birth weight (kg)	0.79 (0.62–1.02)	0.05	0.58 (0.31–1.08)	0.08	0.64 (0.48–0.84)	0.036
Gender						
Female	1					
Male	1.47 (0.98–2.19)	0.06	1.50 (0.96–2.37)	0.07		
Foetal heart rate						
Normal	1					
Abnormal	1.30 (0.73–2.34)	0.36	1.20 (0.63–2.36)	0.55		
Amniotic fluid colour						
Normal	1		1			
Thick meconium	1.43 (0.87–2.36)	0.15	1.48 (0.67–3.26)	0.33		
PPV attempted						
No	1		1			
Yes	1.81 (1.22–2.69)	0.003	1.49 (0.68–3.24)	0.31		
Apgar score 5 minute						
≥7	1		1		1	
<7	4.76 (2.94–7.70)	<0.001	2.81 (1.17–6.70)	0.020	3.20 (1.42–7.22)	0.005
Oxygen saturation on admission						
≥90%						
60–89%	3.04 (1.75–5.28)		3.06 (1.47–6.41)	0.003	3.10 (1.50–6.43)	0.002
<60%	6.36 (3.34–12.10)		4.53 (1.92–10.71)	0.001	4.73 (2.02–11.13)	0.001

PPV, positive pressure ventilation; OR, odds ratio; AOR, adjusted odds ratio; CI, confidence interval; LR, logistic regression. Temperature not included in the model because of missing data

**Table 5:** Logistic regression model of independent predictors of death among term admitted newborns (excluding congenital malformations).

Variables	Bivariate analysis OR (95%CI)	*P-value*	Multivariable analysis with all variables into the model	*P-value*	Multivariable analysis after backward LR elimination	*P-value*
			AOR (95%CI)		AOR (95%CI)	
Foetal heart rate						
Normal	1		1			
Abnormal	1.69 (0.81–3.54)	0.15	1.12 (0.35–3.53)	0.84		
Amniotic fluid colour						
Normal	1		1			
Thick meconium	3.24 (1.68–6.23)	<0.001	2.36 (0.95–5.86)	0.063		
Apgar score 5 minute						
≥7	1		1			
<7	9.11 (5.01–16.58)	<0.001	3.74 (1.44–9.68)	0.006	3.61 (1.67–7.81)	0.001
PPV						
No	1		1		1	
Yes	7.35 (3.09–17.49)	<0.001	4.58(1.23–17.06)	0.023	4.00 (1.40–11.44)	0.010
Oxygen saturation						
<60%	5.8 (2.61–13.21)	<0.001	3.7 (1.24–11.10)	0.019	4.60 (1.82–11.65)	0.001

PPV, positive pressure ventilation; OR, odds ratio; AOR, adjusted odds ratio; CI, confidence interval; LR, likelihood ratio.

An Apgar score <7 at 5 minutes (*p* = 0.005), oxygen saturation on admission (*p* = 0.001) and birth weight (*p* = 0.036) were associated with increased odds of death in the final model for all admitted neonates. Temperature was not included in the model because of missing data points (Table 4). The odds of dying versus survival increased 4.5 fold (*p* = 0.010) with positive pressure ventilation, 3.6 fold (*p* = 0.006) with a 5 minute Apgar Score <7, and 4.6 fold (*p* = 0.001) with an admitted saturation <60% among term newborns (Table 5).

## Discussion

This report highlights the major contributors of neonatal mortality in a low resource rural setting in Northern Tanzania. The findings are consistent with prior reports indicating that BA is the commonest cause of death, with prematurity and its attendant complications, presumed/suspect sepsis, MAS and congenital abnormalities as other important contributing factors. In addition, term newborns who died were 3.7 old more likely to have received positive pressure ventilation in the delivery room, 3.6 fold more likely to have an initial saturation <60% and more likely to present with moderate hypothermia upon admission to the neonatal unit compared to term survivors.

BA and MAS, which were associated with 58% of all deaths, represent intrapartum related complications, likely resulting from interruption of placental blood flow. This number is underrepresented in the present study, due to exclusion of newborns who died in the delivery room within 30 minutes of birth, presumed to be associated with BA [12]. Potential pathways contributing to death in this group included severe hypoxia and moderate to severe hypothermia noted upon admission to the neonatal ward. The severe hypoxia may reflect poor respiratory effort secondary to evolving brain injury (approximately 50% presented with moderate to severe encephalopathy), ventilation/perfusion mismatch (possibly related to MAS or moderate hypothermia) or myocardial dysfunction (possibly related to hypoxia ischemia or moderate hypothermia) [18,19,20]. These newborns were much more likely to evolve to moderate to severe encephalopathy with seizures noted in 30% of cases. The constellation of these clinical findings in the newborns with BA likely contributed to their early death within the first 24 hours, in most infants.

MAS contributed to 10% of all presumed causes of death, which is also higher when compared to the 1.5 to 5% from previous reports [6, 21]. This may represent an over diagnosis due to the inability to obtain chest radiographs, and/or to definitely exclude other pulmonary complications such as pneumothorax, congenital pneumonia and/or concurrent infection. This high mortality rate may reflect the absence of continuous end positive pressure (CPAP) and/or mechanical ventilation, as well as an inability to intubate and potentially administer surfactant, interventions which are regarded as standard of care in developed countries [22].

The above findings offer potential opportunities for reducing mortality related to BA and/or MAS. More than half of newborns who died of BA and MAS had a normal foetal heart rate during labour determined intermittently using a fetoscope in most cases. This raises an important question whether these fetuses could have been identified with continuous foetal heart rate monitoring. The more recent availability of a continuous foetal heart rate monitor termed Moyo, may more readily identify high risk fetuses during labor [23,24]. However our group has recently shown while continuous as opposed to intermittent foetal heart rate monitoring results in the detection of more foetal heart rate abnormalities, it does not reduce the time from detection to delivery, suggesting bottlenecks in the system (i.e. lack of operating rooms and/or skilled clinical providers) [23,24]. Post-delivery, more effective resuscitation including the provision of CPAP and/or endotracheal intubation as indicated above, minimizing hypoxia and maintaining temperature in the normal range are basic first step strategies to potentially reduce early mortality [25, 26]. While both the diagnosis of BA i.e. WHO criteria of a 5-minute Apgar <7 in a non-breathing baby, and that of MAS were made clinically, without evidence of severe fetal acidemia (umbilical cord arterial pH <7.00), multiple organ failure [18,27] or radiological confirmation with MAS, we consider the constellation of clinical findings and potential basic interventions as described above, specific enough to have broad generalizability in potentially reducing early mortality.

Globally, preterm births accounts for 25% of all newborn deaths [6]. In this report, prematurity as the primary diagnosis contributed to 15% of neonatal deaths which is lower than prior Tanzanian and global studies [2, 5, 6]. Preterm newborns who died in this study did not have evidence of intrapartum related complications and their delivery room transitional phase was uneventful. However, more than half of the deaths occurred within the first 24 hours. This is most likely attributed to early respiratory distress due to surfactant deficiency, no CPAP, absence of surfactant replacement therapy and further exacerbated by moderate to severe hypothermia noted upon admission to the neonatal unit [28]. Preterm newborns are highly susceptible to infections and the majority of newborns who died of presumed/suspected sepsis were premature; these infants also had an uneventful delivery room transition. Thus, overall when all diagnoses are combined, prematurity contributed to nearly 30% of deaths in this cohort. Adopting WHO recommended interventions such as administration of antenatal corticosteroids to women who are at imminent risk of preterm labour, antibiotic administration to women with preterm premature rupture of membranes, and initiation of CPAP for newborns with RDS, could improve premature mortality in this setting [9]. In a recent publication from Tanzania, Massawe et al. reported a reduction in mortality in premature newborns of gestational age 28 to 34 6/7 weeks, after administration of antenatal corticosteroids and antibiotics, to both the mother and her newborn [29].

The rate of congenital malformation in this report of 11% is in line with WHO worldwide estimates, as well as the estimates in Tanzania of 14% [5, 6]. The contribution of congenital abnormalities to specific causes of death is probably significantly underestimated in this setting, because of the limited resources to screen with chromosomal analysis or more recent techniques such as microarray and to perform an autopsy after death.

Moderate hypothermia has been linked to an increased risk of death in a dose dependent manner [30]. In this cohort, most admitted newborns with measured temperatures were moderately hypothermic, noted in approximately two thirds of those who died. A recent study from Brazil showed that admission hypothermia was significantly associated with early neonatal deaths even in a presence of good quality newborn care [31]. Continuous improvement of thermal care immediately after birth is critically needed. Potential strategies include early skin to skin care immediately after birth [32], the introduction of overhead heaters or plastic wraps in the delivery room [33] and during transfer of newborns.

From the above it should be apparent that strategies to reduce neonatal mortality in the low-resource setting are complex, influenced by many factors. Reducing mortality due to intrapartum complications will require a multiple prong approach, targeting interventions such as enhanced foetal heart rate monitoring coupled with expedited delivery where indicated, as well as optimizing delivery room resuscitation and in particular, focusing on efforts to maintain the warmth of the newly born. The perinatal management of newborns who are born through thick MSAF requires further study in low-resource countries, with efforts directed towards enhancing respiratory support i.e. CPAP.

The findings of this study should be interpreted cautiously. First, this was a single center study with many resource limitations. Second, this study only included inborn neonates in an area where admitted outborn births are primarily premature neonates, who contribute considerably to mortality in this setting. Third, the challenges of accurate diagnosis without laboratory or radiological studies were substantial, and diagnosis relied mostly on the clinical acumen of the practitioner. Furthermore, postmortem examination to confirm causes of deaths is rare in this setting.

## Conclusion

Intrapartum related complications such as BA and MAS contributed to almost two thirds of the deaths, with prematurity and presumed/suspect sepsis additional important causes. Intrapartum complications and prematurity are the main pathways leading to death in this rural setting. Hypothermia likely played a significant role in increasing mortality in this setting. Strategies to identify fetuses at high risk of intrapartum hypoxia/ischemia, coupled with timely interventions should be a priority in this setting. Implementation of WHO recommended interventions for improving preterm outcome, including improving thermal control and enhancing basic diagnostics such as completed blood count, C-reactive protein and imaging studies is critical to facilitate targeted interventions.

## Supporting information

S1 Dataset Data file. (SAV)
